# Natural variation in early parental care correlates with social behaviors in adolescent prairie voles (*Microtus ochrogaster)*

**DOI:** 10.3389/fnbeh.2013.00021

**Published:** 2013-03-18

**Authors:** Allison M. Perkeybile, Luana L. Griffin, Karen L. Bales

**Affiliations:** Department of Psychology, University of California, DavisDavis, CA, USA

**Keywords:** parental care, natural variation, affiliation, alloparenting, social behavior

## Abstract

Natural variation in early parental care may contribute to long-term changes in behavior in the offspring. Here we investigate the role of variable early care in biparental prairie voles (*Microtus ochrogaster*). Total amounts of parental care were initially quantified for 24 breeder pairs and pairs were ranked in relation to one another based on total contact. Consistency in key components of care suggested a trait-like quality to parental care. Based on this ranking, breeder pairs from the top (high-contact) and bottom (low-contact) quartiles were selected to produce high- and low-contact offspring to investigate adolescent behavior after varying early care. Parental care of subject offspring was again observed postnatally. Offspring of high-contact parents spent more time passively nursing and received more paternal non-huddling contact while low-contact offspring spent more time actively nursing and received more paternal huddling and pseudohuddling in the first postnatal days (PNDs). Low-contact offspring also displayed faster rates of development on a number of physical markers. Post-weaning, offspring were evaluated on anxiety-like behavior, social behavior and pre-pulse inhibition (PPI) to a tactile and an acoustic startle. High-contact offspring spent more time sniffing a juvenile and less time autogrooming. With an infant, high-contact offspring spent more time in non-huddling contact and less time autogrooming and retrieving than did low-contact offspring. Considering sexes separately, high-contact females spent more time sniffing a novel juvenile than low-contact females. High-contact males spent more time in non-huddling contact with an infant than low-contact males; while low-contact females retrieved infants more than high-contact females. In both measures of social behavior, high-contact males spent less time autogrooming than low-contact males. These results suggest a relationship between early-life care and differences in social behavior in adolescence.

## Introduction

There is a large literature in rodents investigating the effects of early manipulations, such as “handling” or “maternal separation,” on offspring development (Levine, 1957; Levine and Lewis, 1959; Denenberg et al., 1962). Brief repeated handling of rat pups in the days just after birth can produce numerous changes in the offspring including a decreased hypothalamic-pituitary-adrenal (HPA) response to non-social stressors (Levine, 1957; Levine et al., 1967; Hess et al., 1969), increased exploratory behavior (Levine et al., 1967; Caldji et al., 2000; Padoin et al., 2001), increased aggression (Padoin et al., 2001; Todeschin et al., 2009), and changes in the oxytocin (OT) system (Noonan et al., 1994; Winkelmann-Duarte et al., 2007; Todeschin et al., 2009). In prairie voles, repeated early handling on postnatal day (PND) 1 increases social behavior toward a novel same-sex conspecific (Boone et al., 2006). A single episode of handling increases exploration in a novel environment (Bales et al., 2007), increases alloparental behavior toward novel infants (Boone et al., 2006; Bales et al., 2007, 2011), and results in formation of a species-typical partner preference, compared to the deficit seen in voles not receiving early handling (Bales et al., 2007). Early handling is often considered to be an enriching experience in that it results in changes in the animal that are typically seen as adaptive (Levine et al., 1967; Fernandez-Teruel et al., 1991; Costela et al., 1993)—they may show more moderate behavioral and physiological responses to stimuli, and greater flexibility in these responses. When compared to this brief early handling, repeated long-term maternal separation during the first few weeks postpartum results in adult offspring that display an increased HPA response to stressors (Plotsky and Meaney, 1993; Ladd et al., 1996, 2004; Liu et al., 2000; Veenema et al., 2006), increased anxiety-like behavior (Ogawa et al., 1994; Boccia and Pederson, 2001; Veenema et al., 2007), increased depression-like behavior (Veenema et al., 2006), and decreased maternal care of offspring (Boccia and Pederson, 2001).

While much of the work on early rodent development features experimental manipulations of offspring or rearing environment, naturally occurring variations in rearing may also produce distinct differences in offspring behavior and physiology. Rat dams display a naturally occurring variation in the amount of care—licking and grooming and arched-back nursing (LG/ABN)—directed toward pups in the first week postpartum (Liu et al., 1997; Francis et al., 1999, 2002; Champagne et al., 2003). High LG/ABN dams produce female offspring that also display high levels of LG/ABN behavior with their own offspring (Francis et al., 1999). Cross-fostering studies have shown that this inheritance is non-genomic in that offspring show patterns of maternal care that are more similar to those of their rearing dam than to those of their biological dam (Francis et al., 1999). These high LG/ABN offspring also display decreased fear behavior in a novel environment (Caldji et al., 1998), a behavior that is, in part, influenced by individual differences in early-life experiences (Francis et al., 1999).

It is clear that experiences early in life can have profound effects on adult physiology and behavioral phenotypes, but most of this work has focused almost entirely on the effects of maternal care. Paternal care, in contrast, is relatively rare in mammalian species, with only approximately 3% of species exhibiting monogamous behavior and, in some but not all cases, extended paternal investment in offspring (Kleiman, 1977). Variation in paternal care can result in changes in offspring behavior similar to that of maternal care as demonstrated through cross-species fostering experiments (McGuire, 1988; Bester-Meredith et al., 1999).

Because biparental care in mammals occurs relatively infrequently across species, its role in shaping offspring outcomes is much less understood than is that of maternal-only care. The prairie vole is a small monogamous rodent originating in the Midwestern United States that forms strong male-female pair bonds and provides biparental care to offspring (Thomas and Birney, 1979; Getz et al., 1981; Williams et al., 1992). This biparental rearing of offspring provides a unique opportunity to investigate the role of not just maternal but also paternal care, or some combination of the two, in shaping offspring physiology and species-typical behavior. It is clear that changes in the environment or brief manipulations of neonatal prairie voles can permanently alter various outcomes for the offspring (Bales et al., 2007, 2011; Ahern and Young, 2009; Stone and Bales, 2010; Stone et al., 2010). There is documented variation in social structure and behavior in the wild, where roughly 40% of males do not form pair bonds with a female, instead taking on a “wanderer” mating strategy (Getz and Carter, 1996). Alloparental behavior also varies greatly—just after weaning most naive female prairie voles display spontaneous alloparental care, but as they reach adulthood this percentage drops drastically until only about 20% of females are alloparental (Lonstein and De Vries, 2000, 2001).

In this study we investigated whether there is variation in parental behavior displayed by pair-bonded prairie voles and if this variation correlates to the later behavior of the offspring. Breeder pairs were initially observed and ranked in relation to one another based on total amounts of care given to offspring. Parental care was then characterized for those pairs ranked as high- and low-contact in the days following the birth of a subsequent litter. Following weaning of this litter, anxiety-like and social behaviors as well as pre-pulse inhibition (PPI) of a startle response were measured in offspring. As this is an initial characterization of natural variation in biparental behavior, we have not used cross-fostering methods and therefore cannot yet determine causation of any difference seen between groups. Based on prior work in similar species, however, we predicted that offspring of high-contact breeders would display decreases in anxiety-like behavior, as measured in an elevated plus maze (EPM) and an open field arena, and would show an increase in pro-social behaviors when interacting with a novel juvenile or a novel infant. These predictions were based on previous work showing that increased early stimulation, either occurring naturally or experimentally, can alter adult behavioral outcomes in rats (Levine et al., 1967; Francis et al., 1999; Caldji et al., 1998, 2000) and in prairie voles (Boone et al., 2006; Bales et al., 2007). We also predicted that high-contact offspring would show greater PPI of their startle response. In rats, early-life handling results in decreased response to a startle stimulus (Caldji et al., 2000). There is also some evidence in primates that rearing with the mother present as compared to peer-rearing leads to a decreased startle response (Parr et al., 2002). It was our expectation that higher levels of early-life stimulation received by offspring of “high-contact” breeders will result in these offspring behaving in a similar manner to the early stimulation models already employed in rats and prairie voles: showing increased sociality toward novel conspecifics and infants, decreased anxiety-like behaviors, and better inhibition of their response to a startle stimulus.

## Materials and methods

### Subjects

Subjects were laboratory-bred prairie voles (*Microtus ochrogaster*), descendants of a wild stock originally caught near Champaign, Illinois. Animals were maintained on a 14:10 light-dark cycle with lights on at 0600. Food (high-fiber Purina rabbit chow) and water were available *ad libitum*. Breeding pairs were maintained in large polycarbonate cages (44 × 22 × 16 cm) and received cotton for nesting material. Upon weaning on PND 20, animals were housed in pairs in smaller polycarbonate cages (27 × 16 × 16 cm). All procedures were reviewed and approved by the Institutional Animal Care and Use Committee of the University of California, Davis.

### Parental care quantification and ranking

Twenty-four breeder pairs were observed to characterize the type and amount of parental behavior directed toward offspring in the first days postnatally. Pairs were observed twice in the morning and twice in the afternoon between PND 1–3 (day of birth is PND 0). Each parent was observed for 20 min while in the home cage; animals were not disturbed during behavioral observations. Males were distinguished from females in each breeder pair based on individual characteristics such as size, fur color and markings, or the presence of pups visibly attached to the nipple. Behaviors were recorded live using behavioral software (www.behaviortracker.com), and included maternal and paternal huddling, pseudohuddling, non-huddling contact, licking/grooming, anogenital licking/grooming, retrievals, hunching, nest building, and autogrooming. In the mother only, lateral, active, and neutral nursing postures were also scored. Because prairie vole pups are born with milk teeth and can attach themselves to the mother's nipples while nursing, mothers can nurse pups while dragging them behind her as she moves around the home cage. This behavior is termed “active nursing” and likely provides a much different experience for pups compared to passive (stationary) nursing, which includes neutral nursing, lateral nursing, and huddling postures. (See Table 1 for ethogram; based on Stone and Bales, 2010).

**Table 1:** **Ethogram of parental behaviors and maternal postures observed during parental care quantification and observations**.

**Parental behaviors**	**Description**
Huddling	All 4 paws touching ground; holding self up over pups; head tucked, back arched
Pseudohuddling	More relaxed huddle posture
Non-huddling contact	In contact with pups and quiescent
Licking/grooming	Licking and grooming pups
Anogenital licking/grooming	Licking and grooming pups, specifically the anogenital region
Retrieval	Lifting pup in mouth and moving it at least 1 in.
Nest building	Moving nest material with either mouth or forepaws
Autogrooming	Grooming self
**Maternal postures**	**Description**
Active Nursing	Pups attached while locomoting around home cage
Lateral nursing	Laying on side with pups laying in front
Neutral nursing	Standing over pups in a relaxed position without locomotion
Hunch	Sitting up on hind limbs in a hunched position; forelimbs off the ground; pups in front

Based on ethograms presented in (Stone and Bales, 2010).

In order to rank breeder pairs in relation to one another, parental care of a single litter was observed from each breeder pair in the colony, with the mother and father as the focal animals. Mean durations of each behavior were computed across the four observations and maternal and paternal means were summed to produce a total parental behavior score for each breeder pair. Scores were then ranked into quartiles based on the amount of total contact directed toward the pups. Parental care of a second litter was then observed in the same manner to determine whether breeder pairs were ranked in the same or an adjacent quartile. Pair rankings fell into the same quartile 92% of the time, suggesting that parental care is trait-like. The top-ranked quartile became the high-contact breeder group while the bottom quartile became the low-contact breeder group. Breeder pairs that fell within the middle quartiles were not used to measure offspring behavior.

### Early parental care of subject offspring

Based on the described quantification and ranking of parental care, we selected the top and bottom quartile of breeder pairs to produce subject offspring. Within 24 h of birth, these pups were removed from the home cage briefly, weighed, sexed, and dyed for identification using Nyanzol dye. If necessary, litters were culled to 4 animals, ideally 2 males and 2 females (high-contact subjects, *n* = 48; low-contact subjects, *n* = 36). Time out of the home cage was kept under 15 min. On PND 1–2 focal observations for each pup were conducted for 20 min in the morning and 20 min in the afternoon (4 observations total) in order to collect detailed data on the type and amount of parental care that was received during the first 2 days postpartum (note that in these observations, the pups were the focal animals). Behaviors were recorded in real-time by a trained observer blind to ranking condition using Behavior Tracker software. All observations were performed while the animals were in the home cage; animals were not handled or disturbed during observations. Pup-directed behaviors were measured from both the mother and father based on the ethogram presented in Table 1.

### Developmental markers

During the first 2 weeks postpartum pups were checked twice daily for the emergence of various markers of development. Observations were made while looking into the home cage; animals were not handled during these checks. Markers recorded included the first day pups were observed with eyes open, eating solid food, leaving the nest under their own volition, and the emergence of fur.

### Behavioral testing

Each subject underwent 6 days of behavioral testing from PND 20–25 (following weaning). Anxiety-like behaviors were recorded from the EPM and the open field. Social behavior was recorded in two situations, a juvenile affiliation test and an alloparental care test. Sensorimotor gating was examined using PPI to both tactile and acoustic stimuli. Except for the PPI testing, all behavioral testing has been previously used and verified in the prairie vole. All testing occurred between 0900 and 1300.

#### Elevated plus maze

Animals were tested in the EPM on PND 20 (high-contact males, *n* = 24; high-contact females, *n* = 24; low-contact males, *n* = 21; low-contact females, *n* = 15) to assess anxiety-like behaviors (Olazabal and Young, 2005). Testing occurred at least 2 h after weaning of the litter. The EPM consisted of two opposing closed arms, with a black floor, black walls, and an open top. Two open arms were set perpendicular to the closed arms; these were open on all sides with a clear Plexiglas floor. Each arm was 67 cm in length and 5.5 cm wide and was elevated 1 m above the floor. At the intersection of the four arms was a center square 10 × 10 cm. At the beginning of each test animals were placed in the center square. Behavior was scored for 5 min by a live observer blind to condition and included time spent in the open arms, time spent in the closed arms, time spent in the center square, autogrooming, and rearing. The ratio of time spent in the open arms/(time spent in the open arms + time spent in the closed arms) was also analyzed. If an animal fell from the open arms of the apparatus, the test was paused, the animal was placed back in the center square and the test was resumed. If an animal fell three times, the test was stopped. The arena was cleaned thoroughly with diluted quatricide between each test.

#### Open field arena

On PND 21 all subjects (high-contact males, *n* = 24; high-contact females, *n* = 23; low-contact males, *n* = 21; low-contact females, *n* = 14) were tested in an open field arena to assess anxiety-like behaviors (Olazabal and Young, 2005). The arena (40 × 40 × 40 cm) was constructed of clear Plexiglas and a grid was marked on the underside of the floor so as to be visible (but not distracting to the animal) during observations. The 10-min test was video recorded and scored later by an observer blind to conditions. Behaviors measured were time spent in the center squares, time spent in the peripheral squares, rearing, autogrooming, and total number of lines crossed. The arena was cleaned thoroughly with diluted quatricide between each test.

#### Juvenile affiliation

Affiliative behavior toward a conspecific was tested on PND 22 (high-contact males, *n* = 24; high-contact females, *n* = 24; low-contact males, *n* = 21; low-contact females, *n* = 15). Adapted from methods described previously (Olazabal and Young, 2005), the testing chamber consisted of two smaller polycarbonate cages (27 × 16 × 16 cm) placed one in front of the other and connected by a short clear tube. Subjects were given 45 min to acclimate to the testing chamber, after which a juvenile (15–19 days old, and collared for identification) was placed into the front cage. Each 10-min test was video recorded and scored later by an observer blind to conditions. Behaviors measured were duration of juvenile-directed behaviors including sniffing, LG, huddling, pseudohuddling, and non-huddling contact, as well as withdrawal, rearing, tumbling, lunging, and autogrooming. Each juvenile was used only once and was then returned to its home cage.

#### Alloparental care

On PND 23 subjects (high-contact males, *n* = 21; high-contact females, *n* = 22; low-contact males, *n* = 21; low-contact females, *n* = 15) underwent an alloparental care test to examine social behavior toward novel infant pups (Roberts et al., 1996, 1998; Lonstein and De Vries, 2001; Bales et al., 2007). The testing chamber consisted of two smaller polycarbonate cages (27 × 16 × 16 cm) placed one in front of the other and connected by a short clear tube. Subjects were given 45 min to acclimate to the testing chamber, after which a novel infant (0–4 days old) was placed in the front cage. Each test was 10 min in duration and was video recorded and scored later by an observer blind to conditions. Behaviors scored included sniffing of the infant, LG of the infant, huddling, pseudohuddling, non-huddling contact, retrievals, autogrooming, and latency to attack. Attacks were very rare. When they did occur, the infant was immediately removed and checked for injury. The infant was treated and returned to its home cage or was euthanized if necessary. Infants were used for a maximum of two tests before being returned to their home cage.

#### Inhibition of a tactile startle response

Subjects were tested on PND 24 (high-contact males, *n* = 24; high-contact females, *n* = 24; low-contact males, *n* = 20; low-contact females, *n* = 15) to assess their ability to inhibit to a tactile startle stimulus following presentation of an acoustic prepulse. PPI testing has not previously been performed in the prairie vole. All parameters used for testing were taken from those used to test several different strains of mice in acoustic and tactile startle inhibition (Paylor and Crawley, 1997). Testing was conducted in an SR-LAB startle box (San Diego Instruments, San Diego, CA, USA). Sessions began by placing the animal into a small Plexiglas cylinder where they were allowed to acclimate for 5 min. Each animal was presented with seven trial types seven times each for a total of 49 trials over 10.5 min. Each trial consisted of a prepulse tone of varying dB followed by a startle air puff. The prepulse tones were 74, 78, 82, 86, or 90 dB followed by a 12-psi air puff startle. There were also two additional trials to measure baseline startle response and baseline activity. Baseline startle response was recorded during a trial with no prepulse followed by a 12-psi startle. Baseline activity was measured during a trial containing no prepulse and no startle. For each trial, a 20-ms prepulse was presented. The startle stimulus was presented 100 ms after the onset of the prepulse and lasted for 40 ms. At the onset of the startle stimulus the subject's startle response was recorded every 1 ms for 65 ms, for a total of 65 response recordings per trial. Intertrial intervals ranged from 10 to 20 s. Each of the seven trial types was presented in a pseudorandom order, with each trial type appearing once in a block of seven trials. Animals were considered to have inhibited their startle response during a trial including a prepulse tone if their percent inhibition from the baseline startle response trial was greater than 40%.

#### Inhibition of an acoustic startle response

The following day subjects (high-contact males, *n* = 22; high-contact females, *n* = 24; low-contact males, *n* = 18; low-contact females, *n* = 15) were run in the startle chambers again to measure the inhibition of an acoustic startle response after an acoustic prepulse. Procedures were identical to those of the tactile startle response except that the air puff startle stimulus was replaced with a 120-dB acoustic tone. Animals completed seven sets of seven trials presented in pseudorandom order.

### Data analysis

For all behavioral data analyses, residuals were checked for normality and, when necessary and possible, were transformed using either a square root or quad root transformation. Not all data could be transformed to a normal distribution. However, there is evidence that analysis of variance (ANOVA) statistics are resistant to non-normality (Feir-Walsh and Toothaker, 1974). In all, seven variables could not be transformed to a normal distribution (EPM: time in the closed arm; open field arena: rearing; juvenile affiliation: rearing, tumbling, lunging; alloparental care: huddling, pseudohuddling). Of these, only one (lunging behavior) reached a trend toward significance. All significance levels were set at *p* < 0.05. For all behavioral data collected, inter-observer reliability was ≥ 95%.

#### Behavior of ranked breeder pairs

**Figure 1:**
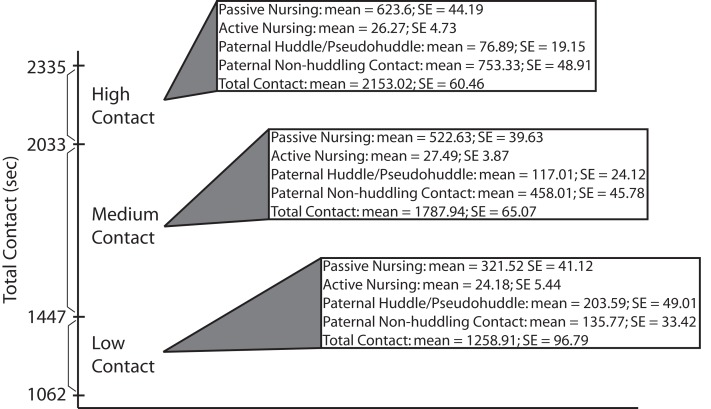
**Breeder ranking data.** Total pup-directed contact time (in seconds) for high-, medium-, and low-contact breeder pairs. Means and standard errors are also presented for specific parental behaviors found to be significantly different between high- and low-contact breeder pairs during early parental care observations of subject offspring (data presented in Figure 2).

Mean durations of each maternal and paternal pup-directed behavior from four observations were summed to produce a “total contact” score for each pair. Scores for each pair were then ranked in relation to one another and those pairs falling into the top (high-contact) and bottom (low-contact) quartiles were selected to produce additional litters to be used for all further testing. Means and standard errors of parental care behaviors from ranking of breeder pairs are presented in Figure 1.

#### Parental care received by subject offspring

Following rank-ordering of breeders on “total contact” behavior, additional offspring were generated as subjects. For these litters, we analyzed differences between the groups in the component behaviors, both to verify the continued validity of the groups and to more carefully investigate in which behaviors the groups differed significantly. Statistical analysis comparing the “high-contact” and “low-contact” groups used a mixed-model ANOVA with group as a fixed variable and the parental breeder pair included as a random variable in SAS 9.2. A false discovery rate correction was done to correct *p*-values for multiple comparisons (Benjamini and Hochberg, 1995).

In order to reduce the number of variables and to determine if there were groups of behaviors that contributed to the differences seen in high- and low-contact parents, a principal component analysis was conducted using the early parental care data. The principal axis method with an oblique rotation was used to extract components. A scree test was used to evaluate the extracted components, of which three were retained, accounting for 38.5% of the variance. For each of the extracted components, a component score was calculated for each individual infant by summing the duration of behavior variables comprising the component. These scores then allowed for comparison of groups to determine if there were differences in the type of care received by high-versus low-contact offspring.

#### Developmental markers

Markers of offspring development were analyzed using a mixed-model ANOVA with parental breeder pair included as a random variable. The ANOVA was performed first with the entire data set and then separately for each sex.

#### Offspring behavior

Data from tests measuring anxiety-like and social behaviors were analyzed using a mixed-model ANOVA with parental breeder pair included as a random variable. These ANOVAs were performed first with the entire data set and then separately for each sex. PPI data were analyzed using a mixed-model ANOVA for each of the five tactile and five acoustic trial types separately with group as a fixed variable and the animal identification included as a random variable. For PPI, the ANOVAs were also performed first with the entire data set and then separately for each sex. In all instances the theoretical interests were in the effects of early contact for each sex. In addition, the combination of two factors (group and sex) in one model can result in an inhibition of each other's effects (Sokal and Rohlf, 1981). For this reason, and as running the sexes separately will result in the same expected means for treatment, the data were analyzed using two separate sex-specific models.

In an effort to reduce the number of variables from measures of offspring behavior into meaningful subsets, principal component analyses were again preformed using data from measures of anxiety-like behavior (EPM and open field arena) and social behavior (juvenile affiliation and alloparental care). The analyses were done following the same methods used for early parental care data and accounted for 41% of the variance in anxiety-like behavior and 36% of variance in social behaviors.

#### Additional analyses

To examine the relationship between early-life parental care and later behavioral in the offspring, a Pearson product-moment correlation was used. Extracted components from the parental care principal component analysis as well as the components extracted from the principal component analyses of offspring behavior were included in the correlation.

## Results

### Parental care quantification and ranking

Means and standard errors for parental care behaviors collected during ranking (in which the breeders were the focal animals) of low-, medium-, and high-contact breeder pairs (*n* = 24) are detailed in Figure 1. Mean total contact durations for the bottom quartile breeder pairs ranged from 1062 to 1447 (mean = 1258.91; *SE* = 96.79) seconds of pup-directed contact. Pairs in the middle two quartiles had durations ranging from 1561 to 1990 (mean = 1787.94; *SE* = 65.07) seconds of pup contact. Durations for the breeder pairs in the top quartile ranged from 2033 to 2335 (mean = 2153.02; *SE* = 60.46) seconds of pup-directed contact.

### Early parental care of subject offspring

**Figure 2:**
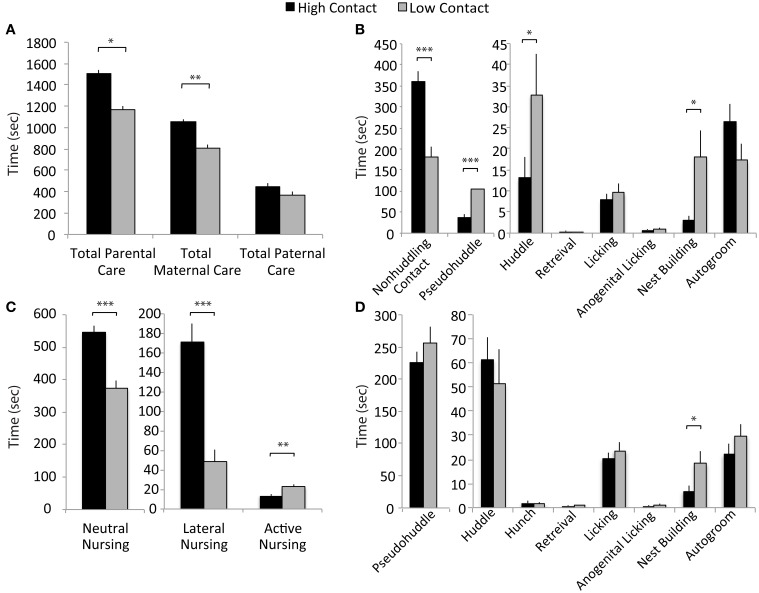
**Early parental care data. (A)** High-contact offspring received more total parental care than did low-contact offspring [*F*_(1, 228)_ = 3.69, adjusted *p* = 0.05]. There was an increased amount of total maternal care directed toward high-contact compared to low-contact offspring [*F*_(1, 228)_ = 9.51, adjusted *p* = 0.002]. **(B)** Fathers of low-contact offspring spent more time huddling [*F*_(1, 228)_ = 5.47, adjusted *p* = 0.04] and pseudohuddling [*F*_(1, 228)_ = 19.87, adjusted *p* = 0.0004] over pups, while high-contact fathers spent more time in non-huddling contact [*F*_(1, 228)_ = 30.24, adjusted *p* = 0.0004]. Low-contact fathers also spent more time nest building [*F*_(1, 228)_ = 6.71, adjusted *p* = 0.02]. **(C)** Mothers of high-contact offspring spent more time in passive nursing postures [lateral nursing *F*_(1, 228)_ = 29.17, adjusted *p* = 0.0006] and neutral nursing [*F*_(1, 228)_ = 32.06, adjusted *p* = 0.0006] while low-contact offspring received more active nursing [*F*_(1, 228)_ = 8.45, adjusted *p* = 0.01]. **(D)** Mothers of low-contact offspring spent more time nest building [*F*_(1, 228)_ = 5.88, adjusted *p* = 0.04]. ^*^p < 0.05, ^**^p < 0.01, ^***^p < 0.001.

Based on results from the ranking of total parental care presented in Figure 1, the breeder pairs in the high- and low-contact groups were selected to produce subject offspring for use in examining behavior post-weaning. These subsequent litters were then observed to better characterize early-life parental care (high-contact subjects, *n* = 48; low-contact subjects, *n* = 36). Observed variations in parental behavior suggest that there are both quantitative and qualitative differences in key components of early-life care of offspring between high- and low-contact breeder pairs, including passive nursing postures and components of paternal care. As expected, across four early-life observations, high-contact subjects received significantly more total parental contact during four early-life parental care observations compared to low-contact subjects [*n* = 304, *F*_(1, 228)_ = 3.69, adjusted *p* = 0.05; Figure 2A]. This was driven primarily by an increase in total maternal care for high-contact offspring [*F*_(1, 228)_ = 9.51, adjusted *p* = 0.002] while there was no difference between groups in the total amount of paternal care given. Although high-contact offspring received greater amounts of total care, low-contact offspring received more of some paternal pup-directed behaviors. Compared to high-contact fathers, fathers of low-contact offspring spent more time huddling [*F*_(1, 228)_ = 5.47, adjusted *p* = 0.04] and pseudohuddling [*F*_(1, 228)_ = 19.87, adjusted *p* = 0.0004] over pups, while high-contact fathers spent more time in non-huddling contact with them [*F*_(1, 228)_ = 30.24, adjusted *p* = 0.0004; Figure 2B]. Similar results were also seen in nursing behaviors—high-contact offspring spent more time in two passive nursing postures [neutral nursing, *F*_(1, 228)_ = 32.06, adjusted *p* = 0.0006; lateral nursing, *F*_(1, 228)_ = 29.17, adjusted *p* = 0.0006; Figure 2C] while low-contact offspring spent a greater amount of time actively nursing, or nursing while the mother was locomoting [*F*_(1, 228)_ = 8.45, adjusted *p* = 0.01]. It is possible that parents of low-contact offspring were engaging in more non-contact parental behaviors as well. Both mothers and fathers of low-contact offspring spent more time nest building compared to parents of high-contact offspring [maternal nest building, *F*_(1, 228)_ = 5.88, adjusted *p* = 0.04, Figure 2D; paternal nest building, *F*_(1, 228)_ = 6.71, adjusted *p* = 0.02].

### Developmental markers

**Figure 3:**
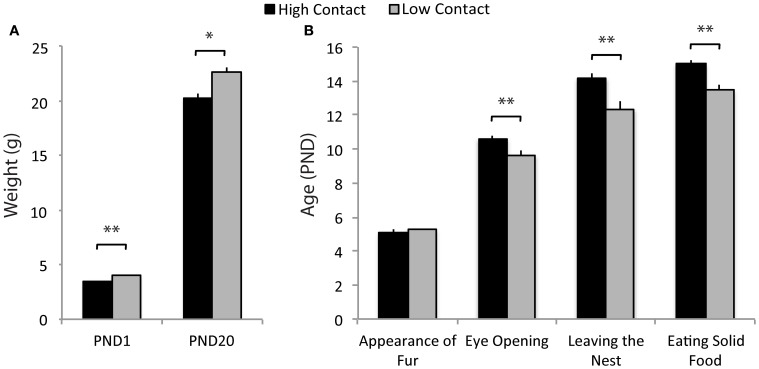
**Developmental marker data. (A)** Low-contact offspring had higher weights on PND1 [*F*_(1, 59)_ = 36.17, *p* < 0.0001] and at weaning [*F*_(1, 63)_ = 10.07, *p* = 0.0023] compared to high-contact offspring. **(B)** Low-contact offspring were observed at younger ages with eyes open [*F*_(1, 55)_ = 31.85, *p* < 0.0001], leaving the nest [*F*_(1, 55)_ = 35.76, *p* < 0.0001], and eating solid food [*F*_(1, 51)_ = 43.70, *p* < 0.0001] than were high-contact offspring. ^*^p < 0.01, ^**^p < 0.0001.

Offspring of low-contact parents developed at a faster rate on a number of developmental markers. Low-contact offspring had higher body weights on PND1 [*n* = 72, *F*_(1, 59)_ = 36.16, *p* < 0.0001; Figure 3A] and on PND20 [weaning; *n* = 76, *F*_(1, 63)_ = 10.07, *p* = 0.0023] compared to high-contact offspring. Low-contact offspring also were observed with eyes open at a younger age [*n* = 68, *F*_(1, 55)_ = 31.85, *p* < 0.0001; Figure 3B], leaving the nest under their own power earlier [*n* = 68, *F*_(1, 55)_ = 35.76, *p* < 0.0001], and eating solid food at a younger age [*n* = 64, *F*_(1, 51)_ = 43.70, *p* < 0.0001] than were high-contact offspring. When sexes were considered separately, there were no significant differences in any measured developmental markers.

### Elevated plus maze

There were no clear differences between groups in measures of anxiety-like behavior when recorded in the EPM with both sexes considered together. We did not find any differences in absolute time spent in either the open or closed arms of the maze, in the total number of arm entries, or in the proportion to time spent in the open arm. Low-contact offspring did spend greater amounts of time in the center of the maze [*n* = 84, *F*_(1, 70)_ = 4.82, *p* = 0.03; Table 2]. When the sexes were considered separately, this difference was seen in low-contact males [*n* = 45, *F*_(1, 31)_ = 4.41, *p* = 0.04] compared to high-contact males.

**Table 2:** **Data from anxiety-like behavior measures**.

**Elevated plus maze**	**High-contact offspring (*n* = 48)**		**Low-contact offspring (*n* = 36)**	
	**Mean**	***SE***	**Mean**	***SE***
Time in center^*^	35.35	4.64	59.92	14.27
Time in open arms	111.10	14.04	103.06	19.41
Time in closed arms	125.56	14.68	142.92	16.90
Time autogrooming	14.92	6.36	21.67	6.61
Total arm entries	11.84	1.16	10.54	1.19
Proportion of time in open arms	0.47	0.05	0.45	0.06
**Open field arena**	**(*n* = 47)**		**(*n* = 35)**	
Time in center	99.76	8.55	93.74	7.60
Time in periphery	493.80	8.16	503.48	7.64
Line crossings	608.21	40.46	613.14	49.69
Rearing	48.80	4.08	49.51	5.72
Time autogrooming	20.31	3.89	21.08	3.80

Elevated plus maze, low-contact offspring spent more time in the center of the maze compared to high-contact offspring [F_(1, 70)_ = 4.82, *p* = 0.03]; Open field arena, groups did not differ in anxiety-like behavior as measured in an open field arena.

* Significant difference between groups; *p* = 0.03.

### Open field arena

There were no differences between contact groups in behavior in an open field arena, either when both sexes were considered together or when each sex was considered separately. Means and standard errors are presented in Table 2.

### Juvenile affiliation

**Figure 4:**
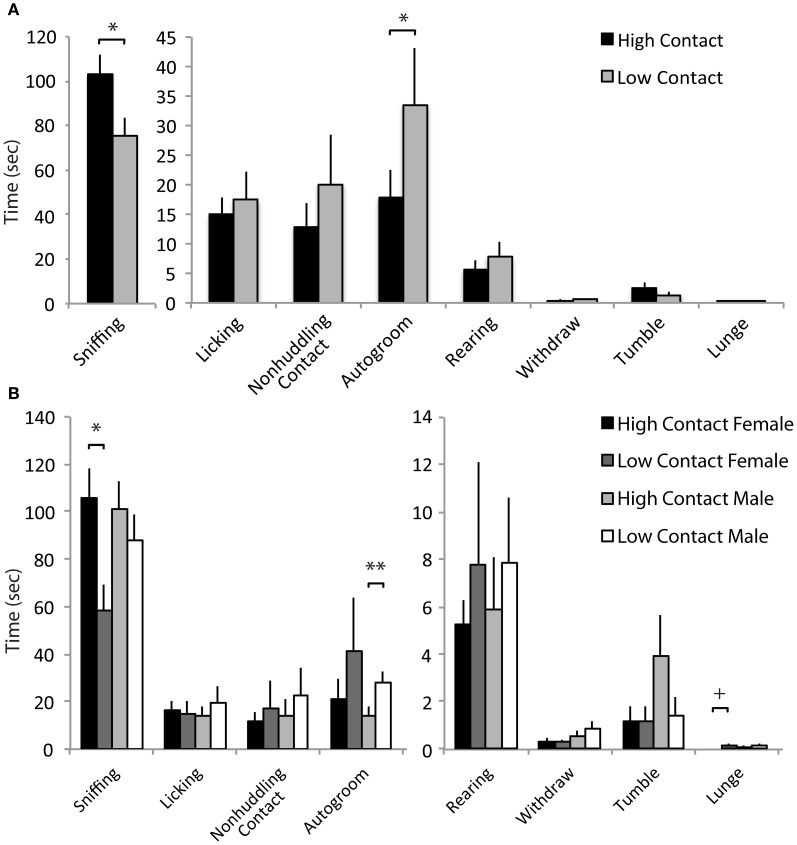
**Juvenile affiliation data. (A)** High-contact offspring spent more time sniffing the novel juvenile [*F*_(1, 70)_ = 5.51, *p* = 0.02] while low-contact offspring spent more time autogrooming [*F*_(1, 70)_ = 4.35, *p* = 0.04]. **(B)** When split by sex, low-contact males spent more time autogrooming [*F*_(1, 31)_ = 5.65, *p* = 0.02]. High-contact females spent more time sniffing [*F*_(1, 25)_ = 7.46, *p* = 0.009] while low-contact females tended to lunge more [*F*_(1, 25)_ = 3.50, *p* = 0.06]. ^*^p < 0.05, ^**^p < 0.01, ^+^*p* = 0.06.

When interacting with a novel juvenile, high-contact offspring displayed greater amounts of prosocial behavior compared to low-contact offspring when both sexes were considered together. High-contact offspring as a group spent more time sniffing the juvenile [*n* = 84, *F*_(1, 70)_ = 5.51, *p* = 0.02; Figure 4A] and high-contact females in particular engaged in more sniffing behavior [*n* = 39, *F*_(1, 25)_ = 7.46, *p* = 0.009; Figure 4B] compared to low-contact females when sexes were considered separately. While high-contact animals showed increased social investigation, low-contact offspring spent more time autogrooming [*F*_(1, 70)_ = 4.35, *p* = 0.04]. This was seen in low-contact males [*n* = 45, *F*_(1, 31)_ = 5.65, *p* = 0.02] when compared to high-contact males. Low-contact females also tended to lunge toward the juvenile more [*F*_(1, 25)_ = 3.50, *p* = 0.06] compared to high-contact females.

### Alloparental care

**Figure 5:**
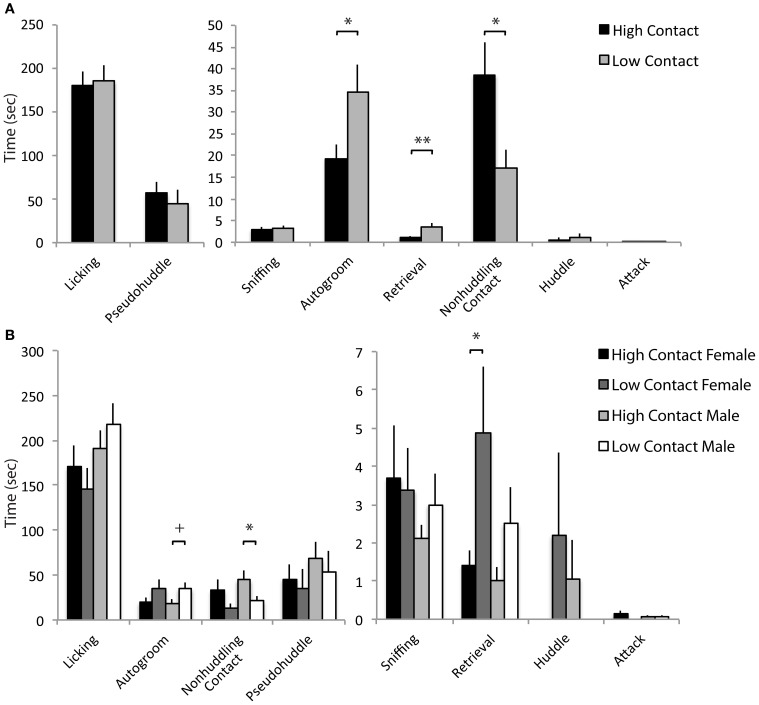
**Alloparental care data. (A)** High-contact offspring spent more time in non-huddling contact with pups [*F*_(1, 66)_ = 5.31, *p* = 0.02]. Low-contact offspring spent more time autogrooming [*F*_(1, 66)_ = 5.21, *p* = 0.02] and retrieved pups more [*F*_(1, 66)_ = 6.59, *p* = 0.01]. **(B)** High-contact males spent more time in non-huddling contact with infants [*F*_(1, 29)_ = 4.07, *p* = 0.05] while low-contact males tended to spend more time autogrooming [*F*_(1, 29)_ = 3.47, *p* = 0.07]. Low-contact females retrieved infants more often [*F*_(1, 24)_ = 5.01, *p* = 0.03]. ^*^p < 0.05, ^**^p = 0.01, ^+^*p* = 0.07.

Increased early-life parental care was related to increases in alloparental behavior toward a novel infant. Here, high-contact offspring spent an increased amount of time in non-huddling contact with a novel infant [*n* = 79, *F*_(1, 66)_ = 5.31, *p* = 0.02; Figure 5A] when sexes were considered together. When sexes were considered separately, this was evident in high-contact males [*n* = 41, *F*_(1, 29)_ = 4.07, *p* = 0.05; Figure 5B] compared to low-contact males. As in the juvenile affiliation test, low-contact offspring as a group spent more time autogrooming [*F*_(1, 66)_ = 5.21, *p* = 0.02], although when exposed to the infant there was only a trend for low-contact males to autogroom more than high-contact males [*F*_(1, 29)_ = 3.47, *p* = 0.07]. Low-contact offspring also retrieved the infant more than high-contact offspring [*F*_(1, 66)_ = 6.59, *p* = 0.01], and this occurred in low-contact females [*n* = 38, *F*_(1, 24)_ = 5.01, *p* = 0.03] but not in high-contact females.

### Tactile startle response

**Figure 6:**
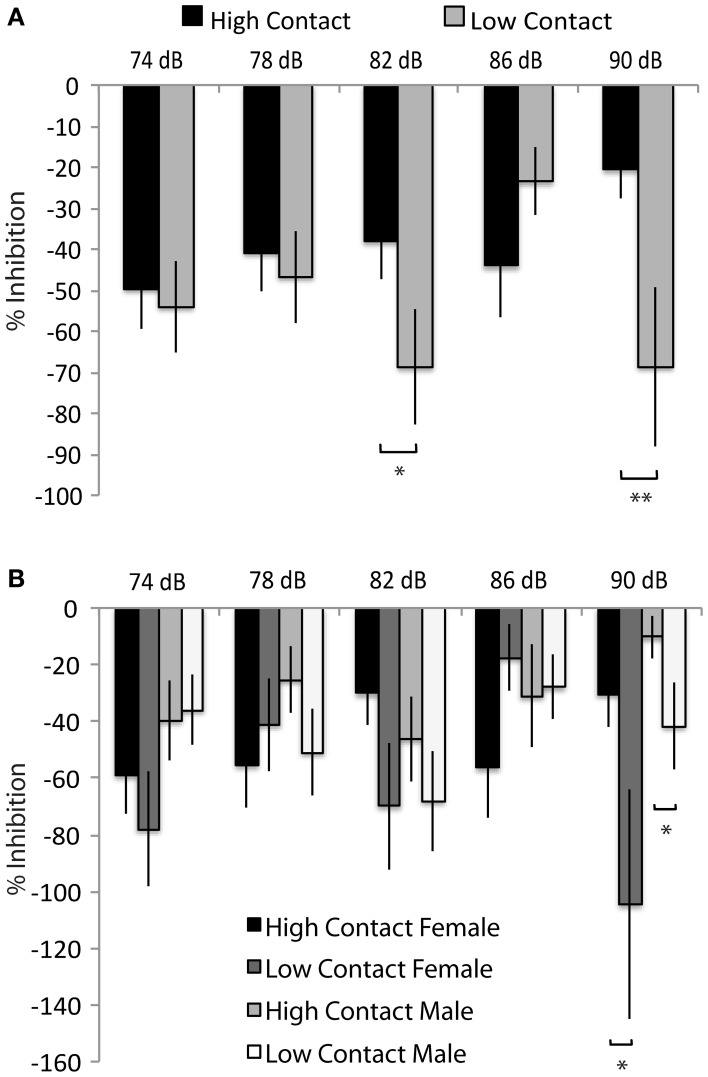
**Prepulse inhibition of tactile startle stimuli. (A)** Low-contact offspring showed a greater potentiation of a tactile startle during tactile Trials 3 [*F*_(1, 468)_ = 3.85, *p* = 0.05] and 5 [*F*_(1, 468)_ = 6.45, *p* = 0.01] compared to high-contact offspring. **(B)** Low-contact males potentiated their startle response more than high-contact males during tactile Trial 5 [*F*_(1, 468)_ = 4.57, *p* = 0.03] and low-contact females potentiated their startle response more than high-contact females during tactile Trial 5 [*F*_(1, 468)_ = 4.05, *p* = 0.04]. ^*^p < 0.05, ^**^p = 0.01.

Both high- and low-contact offspring showed a potentiated startle response to a tactile stimulus instead of the expected inhibition—instead of showing a decreased response to the startle tone following the prepulse, subjects displayed a greater response. When sexes were considered together, low-contact offspring showed an increased prepulse potentiation with an 82 dB prepulse [*n* = 78, *F*_(1, 468)_ = 3.88, *p* = 0.04] and with a 90 dB prepulse [*F*_(1, 468)_ = 6.45, *p* = 0.01; Figure 6A] compared to high-contact offspring. Low-contact male offspring showed an increased startle potentiation with a 90 dB prepulse [*n* = 41, *F*_(1, 468)_ = 4.57, *p* = 0.03; Figure 6B] compared to high-contact male offspring when sexes were considered separately. The same was seen in females, with low-contact females showing more potentiation than high-contact females [*n* = 37, *F*_(1, 468)_ = 4.05, *p* = 0.04]. There were no differences in either baseline activity or baseline startle response, either when both sexes were considered together or when each sex was considered separately.

### Acoustic startle response

There were no differences between contact groups in inhibition of an acoustic startle response, either when both sexes were considered together or when each sex was considered separately. Also, no differences were seen in baseline activity or baseline startle response.

### Early parental care principal component analysis

From the initial analysis of early-life parental care, it became clear that there were both quantitative and qualitative differences in offspring care, so a principal component analysis was done to determine if parental care variables characterized during early-life observations could be reduced into meaningful subsets that could then account for differences in offspring care and relate to differences in behavior. Data for all 19 parental care variables (presented in Table 1) from 14 breeder pairs were included (high-contact breeder pairs, *n* = 8; low-contact breeder pairs, *n* = 6). Factor patterns for varimax and oblique rotations produced similar results. The analysis yielded three components that fit the criteria of the scree test (Hatcher, 1994). Component 1, which we termed passive parental care, included the positively loading behavioral variables of maternal neutral nursing, maternal lateral nursing, maternal pseudohuddling, maternal autogrooming, maternal licking, paternal non-huddling contact, and paternal autogrooming, all of which typically occurred in the nest while the parent is in a relaxed, quiescent state. Component 2, labeled active parental care, included the positively loading variables maternal retrieval, maternal nest building, and paternal retrieval, all of which often resulted in movement of offspring within and out of the next. Component 3, labeled paternal care, included the positively loading variables paternal huddling, paternal pseudohuddling, and paternal licking (see Table 3).

**Table 3:** **Factor loadings for extracted parental behavior components**.

**Variable**	**Factor 1**	**Factor 2**	**Factor 3**
Neutral nursing	0.85		
Lateral nursing	0.46		
Maternal pseudohuddle	0.64		
Maternal retrieval		0.91	
Maternal licking	0.49		
Maternal nest building		0.57	
Maternal autogrooming	0.52		
Paternal huddling			0.72
Paternal pseudohuddle			0.71
Paternal non-huddling contact	0.71		
Paternal retrieval		0.82	
Paternal licking			0.51
Paternal autogrooming	0.61		

Factor loading of <0.4 have not been included. The three factors account for 39% of the total variance.

**Figure 7:**
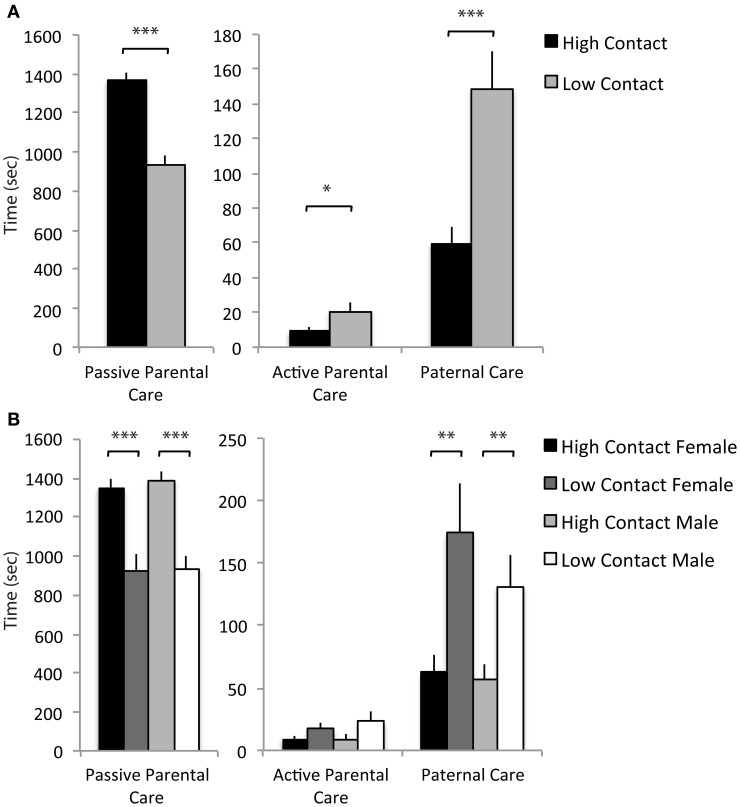
**Parental care components. (A)** High-contact breeders had higher scores for passive parental care [*F*_(1, 228)_ = 56.09, *p* < 0.0001] while low-contact breeders had higher scores for active parental care [*F*_(1, 228)_ = 5.07, *p* = 0.02] and paternal care [*F*_(1, 228)_ = 16.29, *p* < 0.0001]. **(B)** High-contact male offspring had higher scores for passive parental care compared to low-contact male offspring [*F*_(1, 228)_ = 33.68, *p* < 0.0001] and had lower scores for paternal care [*F*_(1, 228)_ = 7.18, *p* = 0.008]. The same paternal was seen in female offspring [passive parental care, *F*_(1, 228)_ = 22.52, *p* < 0.0001; paternal care, *F*_(1, 228)_ = 9.82, *p* = 0.002]. ^*^p < 0.05, ^**^p < 0.01, ^***^p < 0.0001.

Based on the three extracted components, a summed scale was created using raw data for each offspring which included the duration of each of the parental care behaviors comprising the given component. These summed scales were then used in comparisons between high- and low-contact groups and confirmed that there are qualitative differences in the type of care offspring receive. High-contact breeders had higher passive parental care scores [*F*_(1, 228)_ = 56.09, *p* < 0.0001; Figure 7A] while low-contact breeders had higher scores for active parental care [*F*_(1, 228)_ = 5.07, *p* = 0.02] and paternal care [*F*_(1, 228)_ = 16.29, *p* < 0.0001]. There was an increase in passive parental care in high-contact males [*F*_(1, 228)_ = 33.68, *p* < 0.0001; Figure 7B] and females [*F*_(1, 228)_ = 22.52, *p* < 0.0001] compared to their low-contact counterparts and an increase in paternal care in low-contact males [*F*_(1, 228)_ = 7.18, *p* = 0.008] and females [*F*_(1, 228)_ = 9.82, *p* = 0.002] compared to high-contact males and females, respectively. These results indicate that while high-contact offspring receive greater amounts of care overall, they consistently receive less of certain care behaviors, particularly paternal care.

### Anxiety-like behavior principal component analysis and correlational analysis

A principal component analysis was again used to reduce anxiety-like behavioral variables into meaningful components. Data from 5 EPM and 4 open field arena variables (presented in Table 2) from 82 subjects were included. The analysis retained two components. Component 1, termed exploratory behavior, included the positively loading EPM variables time in the center and time in the open arms, the negatively loading EPM variable time in the closed arms, and the negatively loading open field variable rearing. Component 2, termed anxiety-like behavior, included the positively loading EPM variable autogrooming, the positively loading open field variable time autogrooming, as well as the negatively loading open field variable time in the center (see Table 4).

**Table 4:** **Factor loadings for extracted anxiety-like behavior components**.

**Variable**	**Factor 1**	**Factor 2**
**ELEVATED PLUS MAZE**		
Time in center	0.63	
Time in open arms	0.77	
Time in closed arms	−0.51	
Autogroom		0.66
**OPEN FIELD ARENA**		
Time in center		−0.65
Rearing	−0.48	
Autogroom		0.66

Factor loading of <0.4 have not been included. The two factors account for 41% of the total variance.

There was a trend for active parental care to be related to changes in exploratory behavior in a group-specific manner. Overall, there were no significant correlations. However, when analyzing groups separately, there was a trend for low-contact offspring between active parental care and exploratory behaviors (*r* = −0.313, *p* = 0.08) but not for high-contact offspring.

### Social behavior principal component analysis and correlational analysis

A principal component analysis was also used to reduce social behavior variables into subsets. Data from 8 juvenile affiliation and 8 alloparental care variables (presented in Figures 4 and 5) from 79 subjects were included. Three meaningful components were retained. Component 1, affiliative behavior, included the positively loading juvenile affiliation variable non-huddling contact and the positively loading alloparental care variables licking and huddling, as well as the negatively loading juvenile affiliation variable rearing and the negatively loading alloparental care variable attacking. Component 2, termed non-social behavior, included the positively loading juvenile affiliation variable autogrooming, the positively loading alloparental care variables autogrooming and sniffing, and the negatively loading alloparental care variables non-huddling contact and pseudohuddling. Component 3, juvenile investigation and play, included the positively loading juvenile affiliation variables sniffing, withdraw, and tumble, as well as the negatively loading juvenile affiliation variable licking (see Table 5).

**Table 5:** **Factor loadings for extracted social behavior components**.

**Variable**	**Factor 1**	**Factor 2**	**Factor 3**
**JUVENILE AFFILIATION**			
Sniffing			0.67
Non-huddling contact	0.50		
Autogroom		0.70	
Licking			−0.44
Withdraw			0.64
Rearing	−0.53		
Tumbling			0.49
**ALLOPARENTAL CARE**			
Sniffing		0.50	
Non-huddling contact		−0.48	
Autogroom		0.61	
Licking	0.64		
Huddle	0.50		
Pseudohuddle		−0.40	
Attack	−0.60		

Factor loading of <0.4 have not been included. The three factors account for 36% of the total variance.

The type of early parental care received correlated with social behaviors later in life. Passive parental care was negatively correlated with non-social behavior as an adolescent (*r* = −0.296, *p* = 0.01) and was positively correlated with juvenile investigation and play (*r* = 0.224, *p* = 0.05). Meanwhile, active parental care was negatively correlated with juvenile investigation and play (*r* = −0.229, *p* = 0.04). When high- and low-contact groups were analyzed separately, the negative correlation between passive parental care and non-social behavior was seen only in high-contact offspring (*r* = −0.497, *p* = 0.04).

## Discussion

With this study we were able to demonstrate that prairie voles in established breeder pairs vary measurably in the amount and type of care delivered to offspring in the first few days postnatally and that these differences were trait-like, as they were shown to persist from one litter to the next. High-contact offspring received a greater amount of pup-directed parental care overall compared to low-contact offspring. These high-contact offspring also had higher scores for the parenting component comprised of passive parental care, indicating a greater amount of passive and relaxed care received in the early postpartum period. Meanwhile, low-contact offspring had higher scores for the paternal care component. It is unclear whether this increase in paternal care is due to the father taking a more proactive approach to offspring care or the mother spending more time away from the nest or engaged in more non-pup directed behaviors, therefore allowing the father more uncontested access to pups. High-contact breeders also spent more time in passive nursing postures, such as lateral nursing and neutral nursing, while low-contact breeders spent more time actively nursing offspring. These results suggest that while low-contact offspring are receiving a decreased amount of care overall, they in fact receive more of some parental care behaviors than do high-contact offspring.

Low-contact breeding pairs are not simply providing less care for their offspring, they seem to be engaging in a very different *style* of care overall. It appears that parents in high-contact breeder pairs develop a different balance of offspring care between mothers and fathers. Given that total amounts of care are similar within a single breeder pair from one litter to another it may be that this division of parental labor is established early in the pairing and takes on a trait-like quality. Since offspring receive a greater overall amount of care from the mother than the father, driven primarily by the fact that they spend a large portion of their time nursing in the postpartum period, the fact that low-contact offspring receive more of certain types of paternal care and substantially less passive nursing suggests that the father may be compensating for decreased maternal involvement.

Offspring of low-contact breeder pairs showed more rapid maturation across multiple markers of development compared to high-contact offspring. This included a heavier birth weight and a heavier weight at weaning, eyes opening at a younger age, leaving the nest earlier, and eating solid food earlier. There is evidence in rats that decreased maternal LG behavior is related to earlier sexual maturity in female offspring (Cameron et al., 2008). One hypothesis for faster offspring development is that environmental cues can alter parental behavior enough to elicit a response in offspring and a change in their phenotype to allow them to adapt to what will likely be their adult environmental conditions (Hinde, 1986). It is possible that decreased parental care as infants in the prairie vole works in a similar fashion, accelerating offspring development to potentially prepare them for a more harsh environment as adults. Alternatively, it also may be the case that decreased parental care is a result of cues from offspring. Given that low-contact offspring have a heavier weight at birth, it may be that parents invest less care in the first days postpartum based on the increased pup size.

Clear differences in exploratory behavior were not seen when offspring were placed in a novel environment. It was predicted that low-contact offspring would spend less time in the center of the open field arena and more time in the closed arms of the EPM, serving as an indicator of an anxiety-like behavioral phenotype, but this was not the case. High- and low-contact offspring did not differ in the amount of time spent in the center versus periphery of the open field or in either arm of the EPM, suggesting that there may not be clear changes in exploratory behavior at this age. This was unexpected given that models of decreased early stimulation, comparable to the low-contact offspring here, have previously resulted in decreased exploration in novel environments (Caldji et al., 1998; Bales et al., 2007). Low-contact animals did spend more time in the center of the EPM. There is evidence that time spent in the center platform represents decision-making processes (Rodgers and Johnson, 1995; Ohl et al., 2001). Juvenile prairie voles spend a decreased amount of time in the center compared to adults (Olazabal and Young, 2005), displaying less conflict in an anxiety-provoking setting. Based on this, our results suggest that high-contact offspring may display decreased anxiety-like behavior in the EPM.

There was an increase in pro-social behavior directed toward a novel juvenile as well as a novel infant in high-contact offspring compared to low-contact offspring as evident in the increase in non-huddling contact as well as licking of the infant and sniffing of the juvenile. The increased autogrooming by low-contact offspring in both social tests also implies a general increase in social anxiety in these animals when faced with novel animals. These findings agree with previous research showing an increase in social behavior following varying early experience (Boone et al., 2006; Champagne and Meaney, 2007; Ahern and Young, 2009). There is evidence that greater amounts of social interaction during postnatal development result in decreased amounts of social aggression and increased affiliative behavior in rodents (Branchi et al., 2006; Veenema et al., 2007; Curley et al., 2009) and in primates (Winslow, 2005; Rommeck et al., 2011), perhaps similar to high-contact offspring here.

Interestingly, it appears that it is not just the amount but also the *type* of care offspring are receiving that relates to their behavior after weaning, particularly as it relates to offspring social behaviors. Increased amounts of early passive parental care was correlated with decreased amounts of non-social behavior when interacting with a novel infant or juvenile as well as with increased amounts of investigation and play behavior with a juvenile. This investigation and play behavior was also related to active parental care, where decreased amounts of early active parenting was correlated with increased investigation and play behavior in offspring. It appears from these relationships between early care and later behavior that passive early care, involving greater amounts of time quiescent in the nest, is related to greater amounts of prosocial behavior toward novel infants and juveniles in offspring after weaning.

Of particular interest in the early parental care data is the differential role the father appears to be assuming between high- and low-contact breeder pairs, namely that low-contact fathers are actually engaging in more care than their high-contact counterparts. While some paternal behaviors are analogous to maternal care, such as huddling, licking/grooming, and retrievals, they do not necessarily serve the same purpose from an offspring perspective (see Kentner et al., 2010 for review). However, changes in paternal care can result in differential outcomes in offspring much like changes in maternal care do. In the California mouse (*Peromyscus californicus*), a monogamous rodent that displays high levels of paternal care, the presence of the father increases the chance of offspring survival in the field (Gubernick and Teferi, 2000). Presence of the father in the nest also increases social contact time with parents and siblings (Vieira and Brown, 2003) and enhances cognitive development in a sex-specific manner in this species (Bredy et al., 2004). Even without direct rearing behavior, male experiences can have lasting effects on offspring. Offspring of C57Bl/6J mice whose fathers experienced chronic social defeat prior to mating showed increases in anxiety- and depression-like behaviors and males showed higher basal CORT levels (Dietz et al., 2011) while offspring of Balb c/J mice whose father showed increased exploratory behavior in an open field arena themselves displayed increased novel environment exploration (Alter et al., 2009). As discussed previously, increased amounts of maternal care in rats in the first week of life result in offspring who are more social and display decreased anxiety-like behaviors in novel environments. Here we provide evidence that the same may be true for a species displaying biparental care. Offspring who received increased amounts of parental care, including both maternal and paternal care, showed increased affiliative and alloparental behaviors post-weaning. It is not yet known if the differences seen in behavior post-weaning are a result of a varying early care environment or due to genetic differences between high- and low-contact breeders. Given that low-contact offspring received greater amounts of paternal care compared to high-contact offspring, it is possible that fathers in low-contact breeder pairs can work to compensate for decreased amounts of maternal care in offspring.

There were differences between groups and between sexes in the ability to inhibit a startle to a tactile stimulus following an acoustic prepulse, although not in the expected manner. PPI occurs when a weakened stimulus is presented just prior to a startle stimulus, where the weakened stimulus decreases the startle response (Graham, 1975). PPI is thought to be a cross-modal reflex where the prepulse and the startle stimulus do not necessarily need to be presented in the same sensory modality for inhibition to occur (Bullock et al., 1997; Paylor and Crawley, 1997). In the case of our results, it appears that with a tactile stimulus the acoustic prepulse may serve to potentiate the stimulus response. In all tactile stimulus trials, the startle response following a prepulse was proportionally greater than the startle response without a prepulse for both high- and low-contact offspring, suggesting a severely impaired PPI. There is some evidence that startle response reflexes to stimuli of different sensory modalities have different genetic regulation mechanisms (Ralph et al., 2001; Torkamanzehi et al., 2008). Since this potentiated response pattern was not seen in trials with an acoustic startle stimulus, it may be that prairie voles show behavioral inhibition more readily when the prepulse is delivered in the same sensory modality as the startle stimulus, i.e., an acoustic prepulse for an acoustic startle and a tactile prepulse for a tactile startle. This was, to our knowledge, the first published attempt to use a PPI test in the prairie vole. Parameters for testing were based on those used for a range of mouse strains (Paylor and Crawley, 1997) that proved to be effective in eliciting an inhibition to a startle response. It is quite possible that these parameters are not effective in the prairie vole in altering acoustic startle. In this study we saw inhibition differences between groups only with the louder prepulse tones, so it may be that increasing the volume of the prepulse would make it more effective. This idea is supported by subsequent testing in our laboratory that has shown an inhibition of an acoustic startle in animals when the prepulse tone was set at 120 dB (Palmer and Bales, unpublished data).

We have demonstrated that relatively subtle and naturally occurring changes in the early rearing environment of the prairie vole can relate to long-term changes in social behavior, although the mechanism for this change is still unclear. A likely candidate is a change in the OT system (see Bales and Perkeybile, 2012 for review). Increased parental care due to natural variations as seen in the rat model (Francis et al., 1999) has been shown to alter the OT system, a system that plays a large role in social behaviors. Given that early exposure to OT has been demonstrated to alter social behavior and other systems later in life (Bales and Carter, 2003a,b) and touch stimulation causes the release of OT in adults (Uvnas-Moberg, 1998), it is very possible that the increased amount of total parental care received by offspring in this experiment served to change receptor binding patterns or alter neuropeptide release in a way that increased affiliative and alloparental behaviors post-weaning.

To summarize, we have shown that there are reliable variations in parental care in the biparental prairie vole and that these variations are correlated with changes in social behaviors post-weaning (see Table 6 for summary). Because this was an initial investigation of naturally varying biparental care in the prairie vole, cross-fostering of offspring between high- and low-contact parents has not yet been performed. Therefore, we cannot yet determine causation of the changes seen in offspring behavior post-weaning, whether they are due to early parental care input, shared genetic differences between parents and offspring, or, likely, some combination. It is also not known if these differences in parental care are heritable. There is considerable evidence from rats that maternal behavior is passed to female offspring through non-genomic means (Francis et al., 1999; see Champagne, 2008 for review). In the prairie vole, there is evidence for transgenerational transmission of the effects of early handling on parental behavior as well (Stone and Bales, 2010). In this study we considered a large number of behavioral variables. Although we used several strategies to reduce Type I error (including variable reduction procedures and corrections for multiple comparisons), it will be necessary and valuable to replicate these findings in follow-up studies utilizing a more limited number of *a priori* hypotheses. It would also be valuable to investigate the role of maternal versus paternal care as well as the differing outcomes between male and female offspring. While maternal and paternal care may act additively, it may be that paternal care is unique in the way that it shapes offspring behavior in prairie voles.

**Table 6:** **Summary of social and anxiety-like behavioral differences between high- and low-contact offspring by sex**.

		**Male offspring**		**Female offspring**	
		**High-contact**	**Low-contact**	**High-contact**	**Low-contact**
Elevated plus maze	Time in center	Decreased	Increased	–	–
Alloparental care	Retrievals	–	–	Decreased	Increased
	Autogrooming	Decreased^*^	Increased^*^	–	–
	Non-huddling contact	Increased	Decreased	–	–
Juvenile affiliation	Sniffing	–	–	Increased	Decreased
	Autogrooming	Decreased	Increased	–	–
	Lunging	–	–	Decreased^*^	Increased^*^

* Trend toward significance.

### Conflict of interest statement

The authors declare that the research was conducted in the absence of any commercial financial relationships that could be construed as a potential conflict of interest.
